# Analyzing the cyberbullying phenomenon on social media from the perspective of students

**DOI:** 10.3389/fpsyg.2024.1458079

**Published:** 2024-12-19

**Authors:** Maria Cristina Bularca, Sergiu Cristescu, Adrian Netedu, Claudiu Coman

**Affiliations:** ^1^Faculty of Sociology and Communication, Transilvania University of Brasov, Brașov, Romania; ^2^Faculty of Philosophy and Social-Political Sciences, University ‘Alexandru Ioan Cuza’, Iasi, Romania

**Keywords:** cyberbullying, social media, social networks, students, witness, victim

## Abstract

**Introduction:**

With the significant increase in the number of social media users, the degree of cyberbullying has also increased in a directly proportional manner. Cyberbullying manifests itself in the commission of psychological abuses, teenagers being the most vulnerable.

**Methods:**

The purpose of our paper was to analyze how the phenomenon of cyberbullying manifests in terms of frequency on social media platforms, while taking into account factors such as gender, and elements related to the behavior/reactions of witnesses and victims. We conducted a quantitative research, while having as an instrument a questionnaire. We sent the questionnaire online on the Facebook group of students from Transilvania University of Brasov, the sample of the research comprising 500 students. The quantitative method used was considered appropriate for the analysis of this phenomenon because through the questionnaire we were able to gain some insights and a more wide perspective regarding students’ perception about cyberbullying and its prevalence on social media.

**Results:**

The results of the research showed that respondents were aware of the existence of the phenomenon of cyberbullying on social media, and that the main forms of online bullying they experienced or witnessed referred to: ridiculing people’s physical or intellectual aspects and features, being verbally abused or threatened, being humiliated by posting sensitive content, being excluded from a group, or having their secrets publicly revealed. There were no differences found in the degree of exposure to online bullying according to gender, but the results revealed that undergraduate students, and younger students were more exposed to cyberbullying than master students and students of older age.

**Discussion:**

The findings also highlight the fact that witnesses have an important role in the cyberbullying phenomenon. Thus, even though most of the respondents declared that they did not get involved when they witnessed cyberbullying, this results are relevant to prevention policies because they emphasize the need to develop educational campaigns regarding cyberbullying and the need to promote ethical behavior online. The results highlight the need of raising awareness and of educating young people about the phenomenon of cyberbullying, and the need of finding appropriate ways in which this phenomenon could be detected and prevented. From a theoretical point of view, the paper contributes to the literature on the forms of manifestation of cyberbullying on social media platforms, from the perspective of students. In the context of the practical implications of our paper, the results highlight the need of including information about the phenomenon of cyberbullying in the education of young people, the need of conducting awareness and prevention campaigns or programs. The paper also highlights the need of developing and implementing instruments on social media platforms, that could detect the improper behavior of users.

## 1 Introduction

The technological advance of the last 10 years has relentlessly offered us new possibilities to interact with each other and, as in any modern society, communication through the Internet or digital means has become the predominant way of interaction that minimizes the effort. Technologies are developing more and more rapidly and are changing the way we operate in modern society. In this regard, new technologies are continuously improving, bringing more and more opportunities to simplify interactions between individuals. Although this rapid advance of new communication technologies brings many advantages, these digital means also generate a series of spaces and environments conducive to the propagation of unwanted behaviours in the sphere where we also frame the phenomenon of Cyberbullying. Next, it represents a digital form of harassment with significant differences in response, personality, form and content. At the level of recent years, the phenomenon of Cyberbullying, due to the prevalence of this ample phenomenon, has become the subject of international interest.

The study has the main role of analyzing and studying the phenomenological aspects related to cyberbullying or the so-called phenomenon of “cyberbullying” and aims to bring more knowledge regarding the way of spreading this phenomenon in the virtual environment, trying to explain the triggering causes and identify the main solutions both to combat and to prevent it by analyzing all at once the history of its occurrence and propagation in what we call social media today. In this regard, the purpose of our paper was to analyze the way the phenomenon of cyberbullying manifests in terms of frequency on social media platforms, following all at once the nature of gender factors as well as the analysis of elements related to the behaviour/reactions of witnesses and victims. Considering the objectives of the research, in our study we explored the role of witnesses and victims in the experience of cyberbullying on social media, we looked at the main forms of cyberbullying encountered by students on social media, we looked at the degree of awareness that students have about this phenomenon, and we also looked at the frequency with which students use social media and if their opinions and experiences differ depending on gender.

In the elaboration of this study, we used the scientific method as a basis for acquiring new knowledge based on tangible evidence. In order to conduct the research, we used quantitative methods, the research instrument being represented by a questionnaire. The answers obtained were subsequently systematized in a database, processed and interpreted using the IBM SPSS version 23 statistical processor. Looking at the novelty element, the current study offers an updated vision regarding the study of the phenomenon of bullying on the Internet in Romania compared to the level of this phenomenon in 2022. In addition, the current study could also be developed and extended by studying the phenomenon of cyberbullying at international level. In our study we took into account the perspective of students from Romania regarding their experiences with cyberbullying and regarding their perception about this phenomenon. Thus, while taking into account the view of a similar study, which argued that in order to try to prevent bullying, is important to “reach a consensus among children and adults” regarding the forms and meaning of cyberbullying (Hellström et al., 2015), through our study we highlight the importance of analyzing cyberbullying from the perspective of young people. In other words, by examining the perception of students about cyberbullying we can have a more clear view on how they perceive and understand this phenomenon. Hence, this perspective is important at the level of Romania, because cyberbullying has been poorly studied at the level of our country, and it can be important at an international level, because it can offer insights into the way students perceive and experience cyberbullying. Moreover, the results obtained through this study, could be later compared to the results of similar international studies which approach cyberbullying from the perspective of students.

In the first phase, the current research seeks to bring an overview of the issues studied and we sought to achieve a clear, conceptual delimitation between the traditional form of bullying and cyberbullying, highlighting the specific features and forms of each concept, thus reaching the idea that these two forms of abuse are not exactly different by only taking into account the space in which they manifest themselves but also by considering the outline they have, and the component characteristics they incorporate. Hence, even though sometimes, cyberbullying is regarded as simply a type of bullying that takes place in the online environment, we argue that cyberbullying has some characteristics that are different from the characteristics of face to face bullying. In line with this view, a previous study (Willard, 2006), argued that in the case of cyberbullying, there is no physical contact between people and usually the identity of the aggressor is unknown. In other words, one of the characteristics that differentiates face to face bullying from cyberbullying is the fact that the aggressor is anonymous. Furthermore, even though to some degree, cyberbullying can manifest online in the same forms it can also manifest face to face, such as gossiping, spreading rumors or humiliating other people, a previous study emphasized the fact in the case of cyberbullying, due to the characteristics of the medium in which it takes places, it can be very difficult to realize and identify the identity of the perpetrators (Law et al., 2012). Thus, the Cyberbullying phenomenon refers to the repeated and intentional behaviour of injury exercised mainly in the online space and it represents a real problem of the modern world focused on advanced communication technologies. This phenomenon manifests itself among adolescents and young people, especially because of the many possibilities in the cyber area. In this regard, our study seeks to expose the predominant propagation mode, to analyze and detail general aspects related to the depth its propagation through social networks (social media) that present the main consequences of a psychosocial and general nature as a negative consequence of this phenomenon. In the context of cyberbullying among university students, the development of technology can facilitate the phenomenon of cyberbullying. Thus, students access and use frequently a wide range of social networks which offer them many option of interacting with one another, such as: direct/private messages, public messages posted on the accounts of other users, or messages sent as a response to certain posts. Moreover, the use of social networks for academic purposes, like having certain online groups for studying and sharing ideas about the projects developed, can extend bullying in the context of social/personal interactions to bullying in the context of the educational process. Hence, the analysis of the cyberbullying phenomenon on social media is relevant because social networks can facilitate the process of bullying in a number of ways. Compared to face to face bullying, on social networks perpetrators can keep their anonymity, the act of shaming people can be done in front of a large audience, but social networks could also help people report the cases of cyberbullying that they have witnessed or experienced.

### 1.1 Purpose and objectives of the research

The current study aims to analyze the way the phenomenon of cyberbullying manifests in terms of frequency on social media platforms, following all at once the nature of gender factors as well as the analysis of elements related to the behaviour/reactions of witnesses and victims. Thus, the specific objectives of the research approach are:

O1. To establish the main form of cyberbullying that the investigated population has most often faced on social media.

O2. To determine which of the genres was more exposed to the phenomenon of cyberbullying.

O3. To establish to what extent the investigated population is aware of the presence of the phenomenon on social media networks.

O4. To determine how often the investigated population uses social media platforms.

### 1.2 Hypotheses of the research

Related to the research conducted we formulated the following research hypotheses:

Hypothesis 1 (H1). The younger the age of the user of social networks, the higher the chance of being harassed online.

Hypothesis 2 (H2). The degree of exposure to bullying on social networks is significantly different according to gender, type of studies and age categories.

Hypothesis 3 (H3). The actions taken by a victim of online harassment against the aggressor are significantly different if the aggression started in the offline environment or directly in the online environment.

## 2 Literature review

### 2.1 Clarifying the concept of cyberbullying

The development of social networks and the advance of GSM (Global System for Mobile Communication) technologies have become natural things in the contemporary society. The new generations of people benefit from quick access to information and are becoming more and more attracted to virtual environments where well-known social media platforms are also found. The development of such platforms has facilitated the construction of a virtual space where individuals are free to create and upload photo-video content, relate and even carry out online orders and purchases. In addition to these main advantages, these platforms can also generate unpleasant experiences where we identify a recent phenomenon, namely, Cyberbullying. “Unlike traditional bullying, cyberbullying is mainly differentiated by form and medium of manifestation” (Betts, 2016, p. 16) and due to the novelty of the phenomenon we encounter a certain lack of specialized materials regarding the concept behind the term. From an etymological point of view, the term “cyberbullying” is defined as “knowingly harming that has a repetitive character and has the main starting point of text messages” (Hinduja and Patchin, 2008, p. 5). From another perspective, the term cyberbullying is also defined as “an act of violence that is well-oriented by an individual or group of individuals using electronic technologies against a defenseless person” (Hinduja and Patchin, 2008, p. 5). Thus, considering the diversity of the ways in which cyberbullying is defined, a previous study describes cyberbullying as a form of using digital tools in order to directly or indirectly target people, and while describing the phenomenon, the study emphasizes the fact that one should take into account the perspective of the perpetrator, the perspective of the victim as well as the perspective of the bystander (Alipan et al., 2020). Often, the term Cyberbullying is related to other terms and explained as a type of abusive behaviour, abuse or harassment. According to other sources, abusive conduct is explained as a “type of behaviour that is intended to injure the victim by causing psychological harm” (Betts, 2016, p. 16).

As for harassment, this is a form of abuse that consists of a “series of actions that are meant to negatively affect the individual or their group of belonging” (Betts, 2016, p.2). From another author’s perspective, the American term “cyberbullying” is also defined as a “violent behavioural type generated through devices by aggressors who seek to cause harm to an individual or group” (Savage and Tokunaga, 2017, p. 11). Currently, cyberbullying has been defined as “harmful and intentional behaviour that is carried out by an individual or group or individuals, which has a repetitive character, using modern digital technology to assault a victim who is incapable of defense” (Juvonen and Gross, 2008). The aggressor is stronger in some respects than the target and this definition works in parallel with the definition of traditional harassment, essentially adding the concept of “digital technology” as the main mechanism by which the harm to the victim is caused. In addition to all these main features of the phenomenon (intentionality, repetitiveness, power imbalance), several researchers have suggested that “anonymity is an additional characteristic that defines the phenomenon of cyberbullying” (Palladino et al., 2017). In the context of the anonymity theory, in a general manner, anonymity can be described as the state of not being able to be identified, or as the state of being unknown (Hite et al., 2014). In this regard, anonymity may contribute to the development of the phenomenon of cyberbullying, because in the online environment, the aggressor can remain anonymous, there is no need for the aggressor to know or to have previous interactions with the victim, and the victim does not possess any physical evidence, such as scars, from the process of bullying (Barlett, 2015). Moreover, previous studies on the matter of anonymity and cyberbullying (Barlett, 2015; Knack et al., 2021), highlighted the fact that in the online environment people feel that they have more anonymity and this perceived anonymity could facilitate the process of cyberbullying.

The overall characteristics of the digital environment can significantly increase the risks of cyberbullying. These are represented by: the size of the potential audience; continuous access to information; the permanence of online content; the ease of copying the material and distributing it widely and the lack of oversight of behaviors on social media platforms.

### 2.2 The main forms of the cyberbullying phenomenon

The presence of individuals in the online environment increases significantly from year to year. Daily, people use gadgets such as mobile devices and other sources of communication and entertainment to facilitate communication and often they turn to social media platforms and text messages without taking into account the possibility of exposure to less obvious risks. Because this alarming phenomenon has spread rapidly in the online environment, specialized studies have stated that cyberbullying manifests itself in a wide range of ways and these must be analyzed and tracked differently depending on its specific implications. A main pioneer in the research of this ample phenomenon has identified and catalogued a wide range of forms specific to this phenomenon of “where we count: Denigration; Exclusion; Harassment; Outing; Inflammation (flaming); Online tracking (Cyberstalking); Impersonation of False Identities (Impersonation) and Deception (Trickery)” (Shariff, 2008, p. 31).

1. Denigration (Denigration). This form of manifestation of the complex phenomenon counts in ridiculing, demeaning or discouraging the individual using digital means and “can be achieved by publishing on social media the photo, video or information sensitive to the victim.” (Bauman, 2014, p. 54).
2. Rejection (Exclusion) is a social form of exclusion of the individual from a group. This type of cyberbullying manifests itself in the online environment, often within chat rooms or groups on social media platforms. Through this type of cyberbullying, it is given to the individual the impression that he/she is not welcomed in that group (Dobre and Enăchescu, 2016, p. 103).
3. Cyberbullying (Harassment) online is similar to the traditional one. This form of manifestation consists of the existence of hostile and disturbing behaviour based on the physical, and intellectual traits or orientations of the victim. “Technological advancement allows aggressors to become increasingly persistent online because they can be protected from anonymity” (Bauman, 2014, pp. 53–54).
4. Disclosure of secrets (Outing). This form of manifestation counts as “the posting by the aggressor of sensitive information for the victim, information that was never meant to be given for distribution” (Lile, 2017, p. 19) in the social media space. Posting the secretes often happens without the consent of the individual and they are meant to ridicule or demean the individual.
5. Inflammation (Flaming). Inflammation consists of “generating conflict situations that manifest themselves in the social media environment by sending and posting threatening content to an individual or group” (Bauman, 2014, pp. 53–54). This form of manifestation reaches its peak in chat rooms and public social media groups or forums dedicated to a particular topic.
6. Online tracking (Cyberstalking). This form of manifestation consists of “the use of digital means to intimidate and generate a general fear about the personal safety of the individual or persons in his immediate proximity” (Bauman, 2014, pp. 53–54). This type of abusive behavior consists of invading the personal space having a repetitive character, the aggressor often using social media platforms to obsessively follow the activity and actions of the victim.
7. Impersonation of false identities (Impersonation). In the online environment, especially on social media platforms, on public discussion forums but within chatrooms, it may be within the reach of the individual to “claim another identity to steal personal data and information as well as to sabotage good relations” (Lile, 2017, pp. 19–20) of communication with people in the vicinity of the victim’s social circle.
8. Cheating (Trickery). This form of manifestation of the phenomenon of Cyberbullying consists of the deception of an individual by an aggressor by tricking the victim in order to disclose personal information, often embarrassing. After that, the aggressor distributes the sensitive information in the social media environment without having the victim’s prior consent. “Often, the aggressor resorts to this form of cyberbullying to intimidate, blackmail, but also to injure the victim through the potential image damage created to him/her.” (Lile, 2017, pp. 19–20).

At the moment, there are numerous forms of manifestation of the Cyberbullying phenomenon and most often, it is noted that these forms are combined by the aggressor to maximize the efficiency of obtaining what he/she originally set out to do. The combination of methods varies depending on the object pursued by the aggressor but also on the typology and characteristics of the victim or group on which this ingratiated form of abuse is committed. To date, there is no specific institution meant to combat and control this form of abuse, and this is shown by the increased rate of Internet users who have been victims of cyberbullying in the last year at international level.

### 2.3 The concept of social media

Today’s social media platforms and applications allow individuals to connect and interact in an easy way and such platforms made possible the creation and development of large virtual communities, something that until the advent of Web 2.0 was not possible.

Currently, perhaps the handiest definition is the one that describes the social media space as “those internet-based interpersonal communication channels that facilitate mass personal communication and encourage interaction between users” (Zurcher et al., 2018, p. 1), the term “social media” being used for the first time in 1994 and during the development of the Internet the first social media platforms were conceived and launched. Over time, both the number of social media platforms and the number of active users have increased significantly, thus making them some of the most important and attractive parts of the Internet that we know today. “Social media” is used in general as a term that describes a variety of online platforms including blogs, social networks, forums, microblogs, and video photo content-sharing networks. Given this wide spectrum of social media platforms, social media applications are also quite diverse and are not limited to instant content sharing. As of January 2021, there are over 130,000 publications that have the term “social media” included in the title. In articles published over the past 20 years, several authors have formulated several quite different definitions of the term “social media” sometimes using alternative terms. Reporting to the level of publications from the last 12 years to the present, the term social media is beginning to be defined in a variety of ways updated and reported in the context of the continuous development of social media platforms and websites.

In the beginning, in the early development of the Internet and social networks at the level of 2010, the term “social media” was defined simply by referring to “that group of internet-dependent applications that are based on web 2.0 technology and that allow the creation, exchange and distribution of user-generated content” (Kaplan and Haenlein, 2010). A year later, in 2011, a more theoretical definition is presented which exhibits several characteristics. According to her, the term “social media” refers to “a construct consisting of seven great characteristics: identity, conversation, sharing, presence, relationship, reputation and group” (Kietzmann et al., 2011).

Analyzing the last years and taking into account the substantiation of the social media space already in its period of maturity, several authors have found and developed four important definitions that are the closest to the social media space that we know today. Thus, the term and concept of “social media” received updated definitions that are related to the continuous advance and development of recent years, where we list: From the perspective of Carr and Hayes (2015), social media “represents those mass communication channels based on internet connection that facilitate connections and interactions between users generating added value through user-generated content.” From the perspective of Miller et al. (2016) the social media environment is “the space between traditional communication and private dyadic communication that gives individuals several degrees of intimacy.” From the perspective of Leyrer-Jackson and Wilson (2018), social media refers to “those websites and digital applications that allow its users to share content…” From the perspective of Kapoor et al. (2018) the social media environment refers to “those networks that encompass various user-led platforms that facilitate the dissemination of content, the creation of dialogue and communication to the general public.” In essence, this is a digital space created by the individual, for the individual and provides an environment conducive to the development of interactions and the creation of personal and/or professional bonds. Thus, looking at the multitude of definitions and approaches, we argue that social media currently refers to any resource conceived and developed to facilitate a mediated connection between individuals or social actors.

### 2.4 Cyberbullying and social media

Nowadays, there are various ways in which aggressors harass their victims. From the traditional (physical) harassment that manifests itself in the neutral spaces of the aggressor and the victim, the technological advance generated by the development of the social media environment, that includes various applications and social networks, has allowed “traditional” harassment to take on a new form through its implications, namely the form of cyberbullying. Cyberbullying means “the intentional act of intimidation used by the aggressor against the victim through social media” (Beran and Li, 2005), and this seemingly out-of-control phenomenon involves “threatening, denigrating, stealing information and content, blackmailing and spreading false rumors in direct relation to the victim targeted by that cyber aggressor” (Brydolf, 2007).

The negative effects of cyberbullying are numerous, and the consequences of cyberbullying “involve poor professional and personal performance, decreased motivation, physical violence, and suicidal behaviours that are often hidden or masked by the victim” (Willard, 2006). From the perspective of Patchin and Hinduja (2006), the phenomenon of cyberbullying is closely related to a series of effects that generate clear negative consequences such as low self-esteem, family problems, academic problems, violence and delinquent behaviour, and yet the most serious consequence is represented by the suicidal behaviour of the victim. While cyberbullying generates some of the riskiest negative effects as in the case of traditional (face-to-face) bullying, “cyberbullying harassment manifests itself in the absence of physical contact and often without knowing the identity of the perpetrator” (Willard, 2006).

These random acts of cyberbullying go far beyond the scope of the action in terms of traditional (face-to-face) harassment because, unlike traditional harassment, “cyberbullying can manifest itself not only in the common spaces frequented by the aggressor and the victim but also in the personal space or in any other place where the technology is available to the average user and thus accessible” (Shariff and Hoff, 2016). Further, several studies started in this regard have suggested that although cyberbullying may occur and manifest less frequently than traditional (face-to-face) harassment, up to 70% of the total number of respondents to these studies conducted in the United States have stated that they have faced several behaviours in recent years that have been based on cyberbullying (Juvonen and Gross, 2008; Wang et al., 2009).

With the development of new communication technologies through social networks and applications, the phenomenon of cyberbullying has spread and rapidly propagated in this environment since the appearance of the first social network in 1997–1998: SixDegrees, for the simple reason that users predominantly use such platforms that have led to a paradigm shift in the way we interact daily. Thus, apart from facilitating communication and interaction between people, social networks have also provided a dangerous environment where, not infrequently, users have become the targets of cyber aggression. In this regard, bullying through social networks is “a form of manifestation of aggressive behaviour coordinated by an individual or, as the case may be, a group of individuals who exercise a negative behaviour repeated over time and which is directed against often defenseless people” (Duffy and Chan, 2019). This type of bullying is clearly distinguished from other forms of deviant behaviour by the simple fact that it is intentional and directed by the aggressor to a safe victim using new social media communication technologies. Often, this form of deviant behaviour was made possible “by the emergence and development of new information technologies that led individuals to spend more and more time on the internet and implicitly on social media sites and networks” (Duffy and Chan, 2019). Hence, in the context of social networks, while studying the phenomenon of cyberbullying on TikTok, a previous study (Sylvain and Talpade, 2024) showed that TikTok creaters often received hate comments on their videos, and most people received comments from other people who are protecting their identity by having profile pictures that do not represent them. Furthermore, in the context of cyberbullying and Snapchat, a previous study focused on analyzing the perception of educators and practitioners in schools, about the use of Snapchat among students and the study emphasized that due to the features of the platform, cyberbullying and acts of harassment could be magnified on this platform (Charteris et al., 2016).

The change in social activities and the transition from the offline environment to the social media platforms has led to the creation of new possibilities for aggressors to track and harass victims and this was clearly because the large number of social media users has automatically generated the creation of numerous profiles through which the aggressors can more quickly identify their victims. In terms of combating these acts of harassment, it is impossible to eradicate this phenomenon as millions of daily social networks interactions are present, and so it is very difficult to monitor and control all interactions that have violated community policies and standards. “This perspective is thus consistent with the theory of the expediency of crime, which states that social change generates new opportunities in terms of crime and deviant behaviour” (Bastiaensens et al., 2014). The theory of the expediency of crime states that “social and technological changes relentlessly produce new opportunities for crime and deviant behaviour” (Duffy and Chan, 2019). Opportunities in this way play a central role regardless of the nature or severity of the crime and this perspective suggests that changes in social activities (moving from offline to online) offer aggressors new possibilities in terms of engaging in actions specific to cyberbullying.

The increase in the number of users creates the right environment for the manifestation of cyberbullying behaviours from the simple fact that aggressors can identify vulnerable people with some ease by viewing the public profiles present on social networks and social media applications (Bowler et al., 2015). Furthermore, in the context of the forms that cyberbullying can take depending on the type of social network used, a previous study showed that Facebook was the platform on which users mostly experienced cyberbullying, in the form of posting mean comments or spreading rumors (Baruah et al., 2017). On Instagram, for example, due to its visual nature, cyberbullying can happen by posting inappropriate images of someone, but similar to the case of Facebook, it can happen through hateful comments, harassment or denigration (Dewi and Seli, 2023).

## 3 Materials and methods

In order to conduct the research, the scientific method was used, which thus represents the typical way in which “science continues to reach an objective, reliable, verifiable and shareable knowledge of reality thus consisting in the collection of empirical data under the guidance of working hypotheses and theories existing and present to empirical knowledge” (Chelcea, 2001). Regarding the research methodology approached, our research is descriptive, therefore, what we intended to achieve was to define, classify, divide and summarize the problem studied through this type of research. Further, according to the methodology of the proposed research, this study takes the form of quantitative research because it aims to generate models, hypotheses and work theories. Thus, in this descriptive research we used the survey method which represents “an indirect method that is used mainly for the determination of personality traits, attitudes or mentalities that cannot be “brought” to the laboratory” (Chelcea, 2001). Thus, we further substantiated the research approach using the survey technique while having as an instrument a questionnaire, which is an important component of the system of methods in the social sciences.

### 3.1 Sample

The population from which we selected the respondents consists of all the students of Transylvania University of Brasov, a total number of 20,284 students according to the last annual report of the university at the level of the year 2022, from all 18 faculties and all the specializations and educational programs (Bachelor, Master, PhD), that are present and active on the Facebook group created by the students: “Univ. Transilvania din Brașov.” As far as sampling is concerned, this represents “the strict selection, from among several sources of information, of the class of documents that is most relevant to the topic of study and the research objectives” (Chelcea, 2001). In this research, we used the simple random sampling meth od (non-probabilistic) or the so-called randomized sampling which is a simple method by which the subjects (individuals) of the investigated population are randomly selected and thus, each individual has the same chance to be selected and included as the respondent of the study.

The research was conducted between March and April 2023, and the volume of the research is represented by 500 respondents: students of Transylvania University of Brasov present and active on the Facebook group where the research tool, the questionnaire, was shared and applied. The respondents included in the study have the following characteristics: 50% were males and 50% were females; 78% of them are undergraduate students and 22% master students; 59% of respondents come from urban living areas and 41% come from rural living areas; 54% of them are students with the age between 18–21 years; 33% with the age between 22–25 years and 13% with the age between 26–30 years; 95% of respondents stated that they hold Romanian citizenship.

The minimum sample size was tested with the G*Power 3.1.9.7 software and was detailed in each hypotheses proposed to be tested below. The interpretation of the results followed the suggestions of a previous study (Faul et al., 2007). For this research we conclude that our sample of 500 respondents was large enough to exceed the minimum size threshold in each case.

### 3.2 The research tool

The research tool used in order to conduct the research is the questionnaire. According to the classification of Chelcea (1998), depending on the content of the information obtained, the tool used is an omnibus questionnaire that addresses several research topics/ objectives and represents the most common type of questionnaire in sociological research. Next, this type of questionnaire is characterized by a large amount of information on social processes and can also encompass and capture interactions or inter-conditioning between them. Analyzing another criterion, namely the type of questions used, this research tool includes a series of pre-coded (closed) questions to which the variants of answer are pre-established and the respondent thus follows the choice of a variant of answer by his/ her opinion.

In addition to this type of questions, in the construction of the questionnaire, we have also introduced a series of open-ended questions to which the answers are not established in advance, to offer to the respondents the opportunity to freely express their opinions. Finally, the third classification criterion proposed by Chelcea (1998) concerns how the questionnaire is applied and in the present case, this is a self-administered questionnaire because the respondents included in the sample answered to the questions by accessing the questionnaire that was posted in the online environment on the Facebook group of our university. Regarding its elaboration, the questionnaire was transcribed/ typed on the Google Forms platform and the responses obtained were exported in the form of an Excel document and imported into the Statistical Package for Social Sciences (SPSS), version 23, made available by International Business Machines Corporation (IBM). The questionnaire can be found in Appendix A.

Given the analysis of the data collected, we used some specific statistical methods, such as chi-square and factor analysis, because these were the appropriate methods which helped us test the hypotheses of the research and implicitly to see if they are confirmed or not. Hence, in our analysis, we included as predictors gender, types of studies and age categories. To test the hypotheses, we used: the Chi-Square Test of Independence (for Hypothesis H1 and H3), a non-parametric Mann–Whitney U test and nonparametric Kruskal-Wallis Test (Hypothesis H2). For the hypothesis H2 we applied a Principal Components Factor Analysis. The results delivered a single factor (with eigenvalue = 3.29 > 1) that explained 41.23% of the variance in responses. With this analysis we defined our statistical cumulative index named ‘bullying index’. Considering the structure of the questionnaire, it includes a total of 24 questions arranged as follows: the questions from the number 21 to 24 are sociodemographic questions, meant to identify the respondents and to describe the respondents through aspects and information related to gender, age, citizenship, environment of residence and level of education. These questions have a number of variants of answer and take the form of closed pre-coded and open-ended questions. The establishment of the frequency values from question 11 to 18 represents the encoding in variables of the 8 dimensions of the Cyberbullying phenomenon and represents items / values measurable on the Likert scale. Question number 10 is a question that encompasses the scale of social distance that takes into account the measurement of social relations between the actors involved in this phenomenon. Question 1 is a closed pre-coded question that has the role of determining the level / awareness of the phenomenon on social media. Question 2 is a question that takes into account the measurement on the Likert scale of the frequency of use of social media by the respondents involved in this study. Question 3 is intended to determine whether the respondent has been a victim of the Cyberbullying phenomenon. Question 4 is related to question 3 and is intended to go into detail on the platform on which the event happened. Question 5 is an opinion question regarding the respondent’s considerations about social media platforms and the pre-availability of the phenomenon studied on them. Towards the end, the questions from number 6 to number 9 are aimed at establishing the main behavioural reactions of the respondents both as victims and as witnesses of the cyberbullying phenomenon.

To summarize the dimensions mentioned through the questions included in the questionnaire, question 1 measures the dimension regarding students’ awareness of the phenomenon on social media, question 2 measures students’ frequency of use of social media. Question 3 refers to the dimensions referring to whether the respondent has been a victim of cyberbullying and question 4 measures the dimension of the platform on which cyberbullying happened. Question 5 measures the dimension of the students’ opinion about the prevalence of cyberbullying on social media platforms. Questions 6 to 10 measure dimensions related to the main behavioural reactions of the respondents both as victims and as witnesses cyberbullying. Questions 11 to 18 measure the dimension referring to forms of cyberbullying. Question 19 measures the dimension referring to a relation between offline and online bullying, and question 20 measures the dimension related to students’ opinion about the ways cyberbullying can be combated. Questions 21 to 24 measure sociodemographic dimensions.

Given the Likert scale used in the context of measuring the forms of cyberbullying, we used a 5 point Likert scale for frequency: 1—never, 2—rare, 3—several times, 4—frequently, 5—very frequently. The scale measuring the perceived degree of bullying has good reliability (Alpha Cronbach = 0.709, number of items = 8). A frequency scale was considered appropriate because the respondents were asked how frequently they have been a victim in a verbal conflict, how frequently they have been harassed or gossiped about on social media, how frequently someone pretended to take their identity online, how frequently they were excluded from social media groups or they have been threatened on social media. In this regard, the Likert scale used helped us identify how often students experienced various forms of cyberbullying.

Furthermore, considering the way the questionnaire was designed in order to measure the experiences of witnesses and victims, we firstly included in the questionnaire a filter questions through which we asked the respondents if they have been victims of cyberbullying. Then, for those who responded positively to this question, we included an additional question referring to the platform on which the cyberbullying took place. Next, the matter of students being victims of cyberbullying was measured through questions 11 to 18, which were supposed to reveal how often students experienced different types of cyberbullying. Then, for the people who were not victims of cyberbullying, we designed questions referring to the types of cyberbullying they have witnessed or seen in the case of other people on social media, questions referring to the way they reacted when they witnessed cyberbullying, to the reasons why people resort to cyberbullying on social media, to the way they would react if they would be victims, or to the people they would speak to if they would experience cyberbullying (questions 6 to 10).

Hence, the questionnaire was pre-tested on a number of 50 students from Transilvania University from Brasov, in order to assure the clear understanding of the open-ended questions and of the close-ended questions. After the pre-testing process, the students confirmed that they did not encounter any difficulties in understanding and answering the questions.

Moreover, taking into account the objectives of our research and the connection between them and questions included in the questionnaire, in the context of the first objective: to establish the main form of cyberbullying that the investigated population has most often faced on social media, questions 11 to 18, refer to the main forms of cyberbullying that the respondents faced on social media. In the context of the second objective: to determine which of the genres was more exposed to the phenomenon of cyberbullying, question 21 regarding the gender of the respondents is relevant, as well as questions 3 and 4. In the context of the third objective: to establish to what extent the investigated population is aware of the presence of the phenomenon on social media networks, question 1 is relevant and in the context of the fourth objective: to determine how often the investigated population uses social media platforms, question 2 is relevant.

For the questionnaire validity and reliability, we follow the suggestions of Fowler (2009). To increase the validity of the questionnaire we documented other research with very close subject and we pre-test our questions with specialists form our departments, other colleagues from other universities. We summarized all the suggestions to estimate to what extent our work tool measures what it set out to measure. For reliability we applied the questionnaire on a random sample of two group of students and we repeat this research after three mounts. We found that there were no significant statistical differences in the test–retest reliability.

## 4 Results

From the beginning, respondents were asked to specify how often they use social media platforms like Facebook, Instagram, Snapchat, etc. Table 1 presents the frequency with which the respondents use social networks.

**Table 1:** Frequency of use of social networks.

	Frequency	Per cent
Not at all/rarely	35	7
Rare	25	5
Neither rarely nor often	65	13
Often	150	30
Very often	225	45
Total	500	100

We can observe from Table 1 that 75% percent of students declared that they used social networks often and very often. The percentage of respondents who do not use social networks is very low.

Another point of interest was the extent to which the respondents are aware of the presence of bullying on the Internet, with a high incidence, first of all, on social communication networks. Not surprisingly, 96% of the respondents stated that the phenomenon exists on social networks (a fact recognized not necessarily from direct experiences). This unanimity confirms that bullying is no longer an unknown phenomenon, it is easy to identify and, at least theoretically, it should be easy to counteract. In this regard, we tested the first hypothesis of our research:

Hypothesis 1 (H1). The younger the age of the user of social networks, the higher the chance of being harassed online.

To begin with, we used the G*Power software to calculate the minimum sample volume in the case of applying the Chi Square test of independence. In our case, for effect size *ω* = 0.3, alpha = 0.05 and df = 2, we obtained a minimum sample size of 172 subjects. This sample represents the minimum number of observations to be able to assume the statistical effect of type I error probability and power.

The results obtained from testing the hypothesis are further presented in Table 2.

**Table 2:** Cross-tabulation between harassment and age categories.

Variables	*N*	Have you been harassed on social media or the internet so far?		*χ* ^2^	df	*p*
		Yes	No			
Age category				60.949	2	0.000
18–21 years	270	185	85			
22–25 years	165	90	75			
26–30 years	65	10	55			
Total	500	285	215			

To test hypothesis 1, we analyzed the association between the age variable and the dichotomous variable regarding the fact of being harassed on the Internet. Starting from the obtained sample, we grouped the respondents into three age categories: 18–21 years old (with reference to students from the first and second year of studies who are at the beginning of their university education), age 22–25 years old (with reference to students finishing their undergraduate studies) continuing with students between the ages of 26 and 30 (referring to students generally pursuing master’s studies). This division of the sample by age was used to see to what extent the three categories of respondents are significantly different in terms of exposure to online bullying. Considering the results from Table 2, we note that the association of the two variables was statistically significant (*χ*^2^ (2) = 60.949, *p* = 0.000). Thus, we can conclude that younger students are more exposed to bullying on social media.

Moreover, if we consider 100% of those who declare themselves harassed on social media and distribute them by age category, we noticed that 64.9% were from those aged 18–25, 31.6% were from the category of those aged 22–25 years old and only 3.5% belonged to the third category, i.e., 26–30 years old. The magnitude of the effect had the value phi = 0.349 (p = 0.000), so a value justifying an association of moderate intensity but statistically significant. The first hypothesis of our research was confirmed. Hence, younger students could be more exposed to bullying on social media due to reasons related to their perception about the content they see online, their perception about the way people talk online with them. In other words, it is possible for younger students to be more sensitive to criticism ore negative feedback, or to interpret some messages as being directed towards them in a negative manner.

Accurately identifying the social networks on which respondents believe they have been victims of cyberbullying is difficult due to the diversity of sites accessed by users, but also because the sources of bullying can be very diverse. On the other hand, the indication of those platforms can be confused with the platforms currently accessed, out of habit. However, the data obtained from our sample regarding the respondents’ perception of being a victim of cyberbullying and of the platforms on which this phenomenon occurs, are presented in Table 3.

**Table 3:** Respondents’ perception about being victims of cyberbullying.

	On which social media platform you have been a victim of cyberbullying? (%)	On which social media platform do you consider the most present phenomenon of cyberbullying? (%)
Facebook	35	57
Instagram	14	25
Snapchat	5	3
Twitter	2	2
Other	4	1
I wasn’t harassed on social media	35	-
Don’t know/no answer	5	9
Total	100	100

Given the results presented in Table 3, we deduce that Facebook and Instagram are the main platforms on which respondents felt that they have been victims of cyberbullying and implicitly the main platforms on which they consider that the phenomenon of cyberbullying mostly takes place. However, this results could also be influenced by the fact that these two platforms are the ones that are used most frequently globally. Hence, a previous study showed that Facebook and Instagram are indeed two of the most used social networks worldwide (Datareportal, 2024). In this context, considering that the interaction of people mostly takes place on these two platforms, there is a higher chance of the phenomenon of cyberbullying to also take place more often on this platforms than on other social networks.

Another situation should be noted: respondents can be victims of cyberbullying (and can declare or not this) and they can also be witnesses of such situations. As we saw in the previous table, 35% of the respondents declare that they were not victims but we should rather take into account that all the subjects questioned can act rapidly for their safety.

Furthermore, Table 4 presents some of the forms of cyberbullying that were witnessed by the respondents.

**Table 4:** Forms of manifestation of bullying on social media platforms.

	Per cent
The victim has been demeaned by ridiculing his physical or intellectual aspects and features	23
The victim was verbally abused and/or threatened by the aggressor on social media	16
The victim was humiliated by posting disturbed/sensitive images and content	15
The victim has been excluded from a group or social circle	12
The victim had his/her secrets revealed publicly on social media	6
The aggressor assumed a false identity to harass the victim	4
The victim was tricked into providing information and content, which led to acts of blackmail	2
I have never witnessed this phenomenon.	22
Total	100

Being a witness or a victim of cyberbullying can determine various behaviors. In this regard, the results from Table 4, showed that most respondents declared that they have seen that people who were victims of cyberbullying were mostly ridiculed with regards to their physical or intellectual aspects and features (23%). Also, other forms of cyberbullying witnessed by the respondents were the one in which the victim was verbally abused or threatened by the aggressor on social media (16%), and the one in which the victim has been excluded from a group or social circle (12%). However, 22% of the respondents declared that they have never witnessed the phenomenon of cyberbullying.

In the context of taking action when witnessing cyberbullying, the list proposed in the questionnaire captures only a part of the possible decisions to be taken in such situations. In this context, by witnesses we refer to the people who witnessed cyberbullying in the case of other people. Thus, considering the diversity of the ways in which cyberbullying is defined. Thus, the courses of action taken in the case were respondents witnessed such situations are presented in Table 5.

**Table 5:** Courses of action if respondents witnessed bullying on social media.

If you witnessed bullying on social media, how did you react?	Per cent
I did nothing	36
I made my opinion very clear to the aggressor	10
I sought out the victim for support/help	8
I got verbally involved in the conflict	7
I objected to the act of harassment	7
I reported the incident	6
I logged out of the platform	5
I have never witnessed this phenomenon	21
Total	100

In the situations where respondents are witnesses or victims of cyberbullying, there are inevitably various ways to act starting from a set of simple questions such as: am I directly involved?; who is the aggressor?; to what extent an escalation of dialogue can be productive?; the legislative provisions related to the use of the Internet are known in detail by any user?; to what extent a whole series of activities on the Internet are considered much more dangerous? etc. All these elements can lead to the idea of a certain lack of motivation to act in cases of harassment on social media networks mainly in the quality of whiteness. In this case, is not surprising that respondents mainly chose ‘not to do anything’ in such situations (36%). Thus, only 10% of the respondents who witnessed cyberbullying got involved by making their opinion very clear to the aggressor, 8% of them sought out the victim for support/help, and 7% of them got verbally involved in the conflict, or objected to the act of harassment.

However, the percentages can be spectacularly different when the respondent can be directly a victim of cyberbullying (in a hypothetical situation). In this case, the courses of action can be completely different as we it can be seen from Table 6.

**Table 6:** Courses of action if you were to be a victim of bullying on social media?

	Per cent
I report the aggressor’s account	30
I confront the aggressor	27
I express and discuss my problem with a friend	15
I ignore the situation;	13
Leave the platform	10
I do not know/ I do not answer	5
Total	100

Given the data presented in Table 6, most respondents declared that if they were to be victims of cyberbullying they would report the aggressor’s account (30%), that they would confront the aggressor (27%), that they would discuss the problem with a friend (15%). However, 13% of them declared that they would ignore the situation and 10% would leave the platform.

Furthermore, it is interesting to emphasise that, our research also showed that harassment situations on social networks are seen by respondents as personal rather than public or within the competence of the authorities: the students declared that in the position of victims, they want to talk with a friend (60%), a parent (19%), a relative (6%) or others, but not with teachers/tutors (just 2%).

Next, we build a statistical index starting with Q11-Q18 items from the questionnaire. These items represent several eight Likert scales that summarize a series of experiences related to cyberbullying. The descriptive values of each of the eight items is presented in Table 7.

**Table 7:** Experiences related to cyberbullying.

	Min	Max	Mean	SD
11.I was involved as a victim in a verbal conflict on a social network.	1	5	1.97	0.855
12.I have been verbally harassed or threatened on social media.	1	5	1.79	0.865
13.I was gossiped about on social media following rumours	1	5	1.57	0.876
14.Someone tried to pretend to be me on such a social network as Facebook.	1	4	1.48	0.769
15.Embarrassing images or sensitive information targeting me have surfaced on social media platforms.	1	5	1.33	0.736
16.I happened to be tricked on a social network, thus revealing embarrassing information about me that later came to light.	1	3	1.22	0.541
17.I was excluded from a group (regardless of its nature) on social media.	1	2	1.66	0.474
18.In the online environment, on social media I received several repeated threats that generated a state of fear.	1	5	1.49	0.901

To all these items we applied a Principal Components Factor Analysis and the results for our sample were reliable (KMO = 0.752, *p* = 0.000). The results delivered a single factor (with eigenvalue = 3.29 > 1) that explained 41.23% of the variance in responses. The descriptive values of our statistical cumulative index named ‘bullying index’, are further presented in Table 8.

**Table 8:** Descriptive values of the ‘bullying index’.

	*N*	Min	Max	Mean	SD
bullying_index	500	9	29	12.51	3.52061
Valid N (listwise)	500				

According to the results from Table 8, the index has values in the interval [9,29] with a mean = 12.51 and represents a useful tool to measure the extent of bullying on the Internet of our respondents. In the context of this index, we formulated the next hypothesis:

Hypothesis 2 (H2). The degree of exposure to bullying on social networks is significantly different according to gender, type of studies and age categories.

### 4.1 Differences according to the gender of the respondents

We used the G*Power software to calculate the minimum sample volume in the case of applying a nonparametric Mann–Whitney U test for two groups. In our case, for effect size d = 0.5, alpha = 0.05 and Allocation ratio = 1, we obtained a minimum sample size of 244 subjects (sample size for each group was 122 subjects).

A Mann–Whitney U test was conducted and we observed that the males and females are not significantly different with the exposure to bullying on social media platforms (U = 30,275, z = −0.610, *p* = 0.542; mean ranks: 254.4 < 246.6). The difference between males and females is not statistically significant. The hypothesis is not confirmed. Thus, the results which showed no significant difference in exposure to cyberbullying depending on gender, might be influenced by the fact that cyberbullying is often characterized by anonymity and perpetrators may choose the victims not on the basis of their gender but on the basis of other factors, such as popularity, hobbies, or activity domain. Even more, because it takes place online, cyberbullying does not rely on force or power, like it happens in the case of face to face bullying. Hence, in the online environment the stereotypes related to man power or to the weakness of females no longer apply.

### 4.2 Differences according to the type of studies

As in the previous case we used the G*Power software to calculate the minimum sample volume in the case of applying a nonparametric Mann–Whitney U test for two groups. In this case, for effect size d = 0.5, alpha = 0.05 and allocation ratio = 0.28, we obtained a minimum of 278 subjects (for sample size group 1 formed by undergraduate’s students) and 78 subjects (for sample size group 2 formed by master students).

A Mann–Whitney U test was conducted and we observed that the undergraduate students (390 respondents) and master students (110 respondents) are significantly different in the exposure to bullying on social media platforms (U = 15.950, z = −4.151, *p* = 0.000; mean ranks: 264.6 < 200.5). The difference between undergraduates and master students is statistically significant: undergraduate students are significantly more exposed to bullying on social media platforms. The magnitude of the effect had the value d = 0.18 so a value justifying a smaller effect size. The hypothesis is confirmed.

### 4.3 Differences according to age categories

In this case if we use G*Power software for the calculation of the minimum sample size we can use the One-way ANOVA procedure for three groups. For effect size d = 0.25, alpha = 0.05 and three being number of groups we obtained a total sample size of 252 subjects.

Because the Kolmogorov–Smirnov test of normality for the dependent variable was significant statistic (*p* = 0.000) we applied a nonparametric Kruskal-Wallis Test to examine the differences in exposure to bullying on social media platforms according to age categories (1. 18–21 years, 2. 22–25 years and 3. 26–30 years old). Significant differences (Chi-square = 60.386, df = 2, p = 0.000) were found among the three categories of ages. The magnitude of the effect had the value d = 0.35 so a value justifying a medium effect size.

In the continuation of the analysis, a Mann–Whitney test was conducted to compare the group ages two by two. We observed that the respondents from the first two groups were significantly different (U = 1387.5, Z = -7.138, p = 0.000; mean ranks: 251.29 > 163.53), the first and the third group were significantly different (U = 5,375, Z = −4.884, *p* = 0.000; mean ranks: 180.59 > 115.69). The last two groups (2. 22–25 years and 3. 26–30 years old) were not different (U = 5,250, Z = −0.253, *p* = 0.800). In conclusion the first group (18–21 year old) was significantly more exposed to bullying on social media platforms compared to the second group (22–25 years old) and the third group (26–30 years old). In the same time the second and the third group are not different in the exposure to bullying on social media platforms. The hypothesis is partially confirmed.

Next, in order to test the third hypothesis of our research, we made an association analysis for the Q9 and Q19 items from the questionnaire.

Hypothesis 3 (H3). The actions taken by a victim of online harassment against the aggressor are significantly different if the aggression started in the offline environment or directly in the online environment.

In this case we used the G*Power software to calculate the minimum sample volume in the case of applying the Chi Square test of independence. This time for effect size *ω* = 0.3, alpha = 0.05 and df = 4, we obtained a minimum sample size of 207 subjects. This sample represents the minimum number of observations to be able to assume the statistical effect of type I error probability and power.

The results obtained from testing the hypothesis are further presented in Table 9.

**Table 9:** Cross-tabulation between courses of action for victims and the type of harassment.

Variables	*N*	The harassment begins in an offline environment		*χ* ^2^	df	*p*
		Yes	No			
Courses of action				22.228	4	0.000
I ignore the situation	65	20	45			
Leave the platform	50	25	25			
I confront the aggressor	135	65	70			
I express and discuss my problem with a friend;	75	20	55			
Report the aggressor’s account	150	40	110			
Total	475	170	305			

To test this hypothesis, we analyzed the association between the dichotomous variable about the fact that the ‘harassment already began in offline’ (if no- the harassment was all the time just online) and the variable that summarise some courses of action due to harassment. If we discuss just about the respondents who were harassed before in off line (considered here 100%) we obtained that just 30% declared that they ignore the situation, 50% want to leave the platform, 48% declared that they want to confront the aggressor, 26.7% intend to report the aggressor’s account and just 26.7% talk with friends about the course of action. All the values until 100% are reserved to the course of action if the harassment is online.

We observed from Table 9, that the association of the two variables was statistically significant (*χ*^2^(4) = 22.228, *p* = 0.000). Thus, we can conclude that the courses of action chosen by a possible victim of on-line harassment are significantly statistically different. For example, the respondents would rather report an account if the harassment did not start in the offline environment. Thus, the respondents would report the account if the harassment started online. Also, most respondents will ignore the situation if the harassment starts directly online. In other words, most respondents will ignore the situation if the harassment does not start in the offline environment. The hypothesis was confirmed (the association was on the low level of intensity phi = 0.216, *p* = 0.000).

Furthermore, in the context of preventing or combating the phenomenon of cyberbullying, when asked about the actions that should be taken in this regard, respondents proposed some solutions which refer to: blocking the account of people who use insults, threats or inappropriate words towards other people, reporting the account or the aggressor to the authorities, informing and teaching people about the existence of this phenomenon so that they could know better how to deal with it if they experience or witness it.

## 5 Discussion and conclusions

The development of social media platforms facilitated the communication and interaction between people, but also led to the development of cyberbullying—bullying in the online environment. In this context, the purpose of our paper was to analyze how the phenomenon of cyberbullying manifests in terms of frequency on social media platforms, while taking into account factors such as gender, and elements related to the behaviour/reactions of witnesses and victims. In this regard, the objectives of the research referred to: establishing the main form of cyberbullying that the investigated population has most often faced on social media, determining which of the genres was more exposed to the phenomenon of cyberbullying, establishing to what extent the students were aware of the presence of the phenomenon on social media networks, and determining how often the respondents use social media platforms.

Considering the objectives of the research, the results showed that most respondents use social networks often and very often, and that the main forms of cyberbullying experienced or witnessed by students on social media platforms are represented by: ridiculing people’s physical or intellectual aspects and features, being verbally abused or threatened, being humiliated by posting disturbed/sensitive images and content, being excluded from a group, or having their secrets publicly revealed. From this perspective, our paper is in line with other studies which described the forms in which cyberbullying is manifested (Shariff, 2008; Bauman, 2014; Dobre and Enăchescu, 2016; Lile, 2017). Moreover, our paper is in line with a previous study conducted on Romanian students (Mureșan, 2020), which showed that respondents declared that they have encountered cyberbullying in the form of spreading rumours, in the form of being banned or excluded from groups or by being humiliated online.

Furthermore, the results showed that most respondents were aware of the existence of the phenomenon of cyberbullying on social media, and the results showed there were no differences found in exposure to bullying on social media according to gender. However, a previous study conducted at the level of students from Romania (Iorga et al., 2022), showed that the respondents believed that females were more prone to being victims of cyberbullying, compared to males. Contrary to the results of our paper, a previous study conducted on Canadian students, showed that cyberbullying was influenced by the gender of the respondents and that same gender bullying was more often experienced than opposite gender bullying. In other words, females felt that they were more bullied by other females and males that they were bullied by other males. Even more, the same study also showed that females reported more negative effects of cyberbullying on their personal life and on their educational performance, compared to male respondents (Faucher et al., 2014).

Given the first hypothesis of our research, the results revealed that younger students (those aged between 18 and 21 years old) tend to be more exposed to online bullying, compared to students aged between 26 and 30 years old. Thus, the first hypothesis was confirmed.

Moreover, in the case of the social networks on which the respondents declared that they have been victims of cyberbullying, or on which they considered the phenomenon to be most present, the main social networks mentioned were Facebook and Instagram. In this regard, our study is in line with a previous research conducted on students in the United Arab Emirates (Abaido, 2020), in which Facebook and Instagram were identified as the main platforms on which cyberbullying takes place. However, this result could also be influenced by the fact that Facebook and Instagram are two of the most used platforms worldwide, and this would mean that the increased frequency of cyberbullying on these social networks could be given by people’s frequency of using them. From this point of view, our paper is in line with previous studies (Bowler et al., 2015; Craig et al., 2020), which stated that together with the increase of the numbers of users of social media platforms, an increase of cyberbullying can also occur, due to the fact that aggressors can identify their potential victims more easily. Even more, our paper is also in line with a previous study which was conducted on young people aged 14 to 17 years old from seven European countries (Athanasiou et al., 2018), which emphasized the fact that in the context of Romania, Poland and Germany, spending more than 2 h per day on social networks was associated with a higher risk of experiencing cyberbullying.

Given the actions that the respondents took in the case in which they witnessed cyberbullying, a rather concerning results showed that most of them declared they did not take any action. In the event in which they took action, some of them actions taken referred to making their opinion clear to the aggressor, getting verbally involved in the conflict, or objecting to the act of harassment.

However, if they were to be victims of cyberbullying, students declared that they would mostly report the aggressor’s account, they would confront the aggressor, or they would discuss the problem with a friend. Even more, most students would discuss the issue with people they hold close, but they would not discuss it with their teachers.

Considering the second hypothesis of our research, the results showed no differences in the degree of exposure to cyberbullying between males and females, but they revealed differences according to their level of study and the age of the respondents. In this regard, undergraduate students were more exposed to online bullying compared to master students, and students aged between 18 and 21 years old were more exposed compared to those aged between 22 and 25 years old or between 26 and 30 years old. Hence, the second hypothesis was partially confirmed.

Considering the third hypothesis, the results revealed that the actions taken by students in the event of bullying are different depending on the environment in which the harassment has taken place (online or offline). Thus, students would report the account if the harassment started online, but they would ignore the situation if the harassment started directly online.

Furthermore, in the context of preventing the development of cyberbullying, some of the solutions proposed by the respondents included: blocking the social media accounts of people who address insults or abuse other people online, reporting their accounts to the authorities or educating people about the existence of the phenomenon of cyberbullying and its consequences. In context of educational measures, our paper is in line with a previous study (Carter, 2013), which emphasized the need for internet safety education and for the education of witnesses or third party observers of online bullying. In the context of solutions which could be taken into account for the prevention of cyberbullying in educational institutions, some digital education programs could be developed, and some new features could be added to the social media platforms, feature which could help with the detection of aggressive behaviour. Even more, institutions could develop mentorship programs for students, in which older students could help younger students to use social media in a more responsible manner and to protect themselves from cyberbullying and its negative effects. Taking into account the theoretical and practical implications of our paper, from a theoretical point of view, the paper contributes to the literature on the forms of manifestation of cyberbullying on social media platforms, from the perspective of students. Given the practical implication of our paper, the results highlight the need of including information about the phenomenon of cyberbullying in the education of young people, the need of conducting awareness and prevention campaigns or programs. Even more, the paper highlights the need of developing and implementing instruments on social media platforms, that could detect the improper behavior of users. Thus, in the context of educators, the results of the study could be taken into account by educators and teachers, and they could be presented to high school students and university students in order to increase awareness about the phenomenon of cyberbullying. Furthermore, policymakers could make use of the findings of our study by looking at the forms of cyberbullying encountered by students, and at the platforms on which this phenomenon is most likely to take place, and they could develop policies which could contribute to diminishing this phenomenon. Next, social platforms could take into account the findings of the research in order to develop policies against cyberbullying on social networks, or in order to create and develop filters/buttons which could help users report bullying or stop user from bullying each other.

### 5.1 Limitations and future research directions

Considering the limitations of our research, one limitation is represented by the fact that only quantitative methods were used in order to conduct the research. In this regard, a future research could take into account using qualitative methods, such as interviews in order to gather information about the phenomenon of cyberbullying. Another limitation is represented by the fact that data was obtained from students only from one university, and in a future research, the opinion of students from other universities from Romania or abroad, could be studied. Moreover, apart from gathering information from students, in a future research, the opinion of decision makers or representatives of authorities could be taken into account, in order to analyze ways in which cyberbullying could be prevented or combated. Other research directions could be represented by analyzing the role of artificial intelligence in preventing cyberbullying, and on comparing cyberbullying with forms of traditional bullying in order to better develop strategies or policies meant to prevent the development of such phenomenons.

## Data Availability

The original contributions presented in the study are included in the article/Supplementary material, further inquiries can be directed to the corresponding author.
