# Supplementary material for: Analyzing the cyberbullying phenomenon on social media from the perspective of students
Source: Front Psychol. 2024 Dec 19;15:1458079. doi: 10.3389/fpsyg.2024.1458079 (PMC11695848; doi:10.3389/fpsyg.2024.1458079)
Supplement: Supplementary file 1 [file Data_Sheet_1.docx]

# Appendix A. Cyberbullying questionnaire

1. Do you think cyberbullying is present on social media?

- Yes
- No

1. On a scale of 1 to 6 where 1 means none and 6 very often, how often do you use social media platforms like Facebook, Instagram, Snapchat, etc.?

- Not at all;
- Rarely;
- Rare;
- Neither rarely nor often;
- Frequently;
- Very often.

1. Have you been harassed on social media or the internet so far?

- Yes;
- Not.

1. If you answered "Yes" to the previous question, on which social media network did the event happen?

- Facebook;
- Twitter;
- Snapchat;
- YouTube
- Instagram;
- Another;
- I have not been harassed on social media.
- 99.I know / I don't answer.

1. From your perspective, on which social media platform do you consider the most present phenomenon of Cyberbullying?

- Facebook;
- Twitter;
- Snapchat;
- YouTube
- Instagram;
- Another;
- 99. I do not know / I do not answer.

1. If you have witnessed bullying on social media platforms, in what form has it manifested itself?

- The victim was humiliated by posting disturbed/sensitive images and content;
- The victim has been excluded from a group or social circle;
- The victim has been demeaned by ridiculing his physical or intellectual aspects and features;
- The victim had his secrets revealed publicly on social media
- The victim was verbally abused and/or threatened by the aggressor on social media;
- The aggressor assumed a false identity in order to harass the victim
- The victim was tricked into providing information and content, which led to acts of blackmail;
- I have never witnessed this phenomenon.

1. If you witnessed bullying on social media, how did you react?

- I made my opinion/vere clear to the aggressor;
- I got verbally involved in the conflict;
- I did nothing;
- I logged out of the platform;
- I objected to the act of harassment;
- We sought out the victim for support/help;
- We reported the incident;
- I have never witnessed this phenomenon.

1. In your opinion, for what reasons do you consider that abusers harass their victims on social media?

- Out of boredom;
- To become popular;
- Because of the presence of a self-defense mechanism for their frustrations;
- Because of personal problems and frustrations;
- 99. I do not know/ I do not answer.

1. What would you do if you are a victim of bullying on social media?

- I ignore the situation;
- Leave the platform;
- I confront the aggressor;
- I express and discuss my problem with a friend;
- Report the aggressor's account;
- 99. I do not know/ I do not answer.

1. If you were to talk to someone about this issue that hypothetically speaking you were involved in, who would you tell them?

- To a friend;
- A teacher/tutor;
- To a parent;
- To a relative;
- Anyone.

On a scale of 1 to 5 where 1 means never and 5 very frequently, set the corresponding value of the following statements:

1. I was involved as a victim in a verbal conflict on a social network.
2. Never;
3. Rare;
4. Several times;
5. Frequently;
6. Very frequently
7. I have been verbally harassed or threatened on social media.
8. Never;
9. Rare;
10. Several times;
11. Frequently;
12. Very frequently
13. I was gossiped about on social media following rumors started on them.
14. Never;
15. Rare;
16. Several times;
17. Frequently;
18. Very frequently
19. Someone tried to pretend to be me on such a social media platform as Facebook.
20. Never;
21. Rare;
22. Several times;
23. Frequently;
24. Very frequently
25. Embarrassing images or sensitive information targeting me have surfaced on social media platforms.
26. Never;
27. Rare;
28. Several times;
29. Frequently;
30. Very frequently
31. I happened to be tricked on a social network, thus revealing embarrassing information about me that later came to light.
32. Never;
33. Rare;
34. Several times;
35. Frequently;
36. Very frequently
37. I was excluded from a group (regardless of its nature) on social media.
38. Never;
39. Rare;
40. Several times;
41. Frequently;
42. Very frequently
43. In the online environment, on social media I received several repeated threats that generated a state of fear.
44. Never;
45. Rare;
46. Several times;
47. Frequently;
48. Very frequently
49. Before I was harassed on social media by a certain person, it first manifested itself in the offline environment (Face to face).

- Yes;
- Not;
- I have not been harassed on social media.

1. In your opinion, how do you think bullying on social media platforms can be combated?

..................................................

1. What gender category do you fall into?

- Male
- Female

1. What is the category of your age?

- 18-21 years old;
- 22-25 years years old;
- 26-30 years old;

1. What is your nationality?

- Romanian;
- Other citizenship.

1. What is your current form of education?

- Undergraduate
- Master
